# Two nonsense somatic mutations in *MEN1* identified in sporadic insulinomas

**DOI:** 10.1002/2211-5463.12366

**Published:** 2018-01-02

**Authors:** Cheng Qi, Jiayue Duan, Qingfeng Shi, Mingguang Wang, Changqing Yan

**Affiliations:** ^1^ Department of Surgical Oncology The Second Hospital of Hebei Medical University Shijiazhuang China; ^2^ Department of Hepatobiiary Surgery The Second Hospital of Hebei Medical University Shijiazhuang China

**Keywords:** insulinoma, *MEN1*, somatic mutation, whole exome sequencing

## Abstract

Insulinomas are functional pancreatic neuroendocrine tumors that cause hypoglycemia and severe morbidity. The aim of our study was to identify gene mutations responsible for tumorigenesis of sporadic insulinoma. Whole exome sequencing analysis was performed on tumors and paired peripheral blood from three patients with insulinomas. After initial analysis, somatic mutations were obtained and a deleterious protein product was further predicted by various bioinformatic programs. Whole exome sequencing identified 55 rare somatic mutations among three insulinoma patients, including *MEN1* gene nonsense mutations (c. 681C>G; p.Tyr227* in exon 4 of *MEN1* and c. 346G>T; p.Glu116* in exon 2 of *MEN1*) in two different tumor samples. The mutations resulted in a significant truncation of the protein and a non‐functional gene product, which was involved in defective binding of menin to proteins implicated in genetic and epigenetic mechanisms. Our results extend the growing list of pathogenic *MEN1* mutations in sporadic cases of insulinoma.

**Abbreviations**
MEN‐1: multiple endocrine neoplasia type 1
SNV: single nucleotide variant

Insulinoma is a rare and sporadically occurring neuroendocrine tumor that secretes an excess of insulin, resulting in symptoms of hypoglycemia in patients 1. Additionally, the catecholamines released from insulinoma produce several symptoms including sweating, nausea, weakness, anxiety and palpitation 2. Insulinoma is usually a benign neoplasm that is smaller than 2 cm in diameter, without signs of angiogenesis or metastases, and is easily curable by surgical resection 2. However, an understanding of the mechanism of the pathology of insulinoma is still needed. The small number of disease cases and lack of suitable animal models and cell strains have limited the study of the pathogenesis of insulinoma. The risk factors and related molecular mechanisms of insulinoma remain unclear.

Exome sequencing, which allows study of the complete protein‐coding regions in the genome, is valuable in searching for underlying genetic variation in disease. Moreover, extensive exome sequencing studies from human tumors have indicated that there are a large number of mutations in each tumor 3. Accumulating evidence suggests that genetic change is specific for insulinomas, such as high loss of heterozygosity rates on chromosome 22q and gain of 9q34 4, 5. Additionally, exome sequencing has revealed that there was a somatic mutation in the DNA‐binding zinc finger of the transcription factor Yin Yang 1 in insulinoma 6, 7, 8.

In order to further screen for potential genetic alterations in insulinoma, we selected the tumor tissue and matched blood of patients with insulinoma and performed exome sequencing and analysis of deleterious effects on the protein. In this study, we obtained a total of 55 gene mutations in insulinoma. Among them, mutations in *MEN1* were most related to the pathology of insulinoma, which provides evidence for use in early disease screening and target treatment of insulinoma.

## Materials and methods

### Insulinoma patient samples

In our present study, three patients (INS1, INS2 and INS3) with primary insulinoma were enrolled from the Second Hospital of Hebei Medical University. The paired tumor and a peripheral blood sample for DNA extraction were collected from the patients after surgical removal. The insulinoma was diagnosed depending on current clinical guidelines and histopathological confirmation 9. The diagnosis was established using the following six tight criteria: (a) documented blood glucose levels ≤ 2.2 mmol·L^−1^ (≤ 40 mg·dL^−1^); (b) concomitant insulin levels ≥ 66 μU·L^−1^ (≥ 36 pmol·L^−1^; ≥ 3 μU·L^−1^); (c) C‐peptide levels ≥ 200 pmol·L^−1^; (d) proinsulin levels ≥ 5 pmol·L^−1^; (e) β‐hydroxybutyrate levels ≤ 2.7 mmol·L^−1^; (f) absence of sulfonylurea (metabolites) in the plasma and/or urine. Written consent forms were obtained from the enrolled participants, and the research protocol was approved by the ethics committee of the Second Hospital of Hebei Medical University (2017‐R086) and complied with the principles of the Declaration of Helsinki. Clinical information for the patients is shown in Table 1.

**Table 1:** Clinical information for the patients with insulinoma. INS1, INS2 and INS3 represent the three patients with insulinoma

Sample	Gender	Age at diagnosis (years)	Grade	Metastatic disease	Tumor size (cm)	Ki67
INS1	Male	64	G2	No	0.6	2%
INS2	Female	75	G1	No	0.8	2%
INS3	Female	57	G2	No	1.0	2%

### Exome sequencing and data analysis

Genomic DNA was isolated from blood and tissue and was controlled for quality by measuring its concentration using a Nanodrop 2000 (Illumina, San Diego, CA, USA) and measuring fragmentation by agarose gel electrophoresis. Qualified Genomic DNA was prepared for exome sequencing with an Agilent SureSelect Human All Exon 50 Mb Exon Kit (Agilent Technologies, Santa Clara, CA, USA). The genomic DNA of each sample was fragmented and captured for exome sequencing with the Illumina HiSeq 2500 Sequencer platform (Illumina). For each sample, sequencing reads with 125‐bp paired‐end and *Q*_30_ > 92% were generated.

After filtering the low quality and contaminating reads, sequence reads were mapped to the human genome sequence (hg19) using the Burrows–Wheeler alignment tool (http://bio-bwa.sourceforge.net/), which generated the sequence alignment/map file. The PCR duplicate reads were further removed using the picard software program.

### Single nucleotide variant detection and annotation

To obtain the important candidate genes, the mutect software 10 was used to detect single nucleotide variants (SNVs). Variants were filtered for minimum genotype quality of 50 and minimum coverage depths of 10. Then, the software annovar (http://www.openbioinformatics.org/annovar/) was applied to annotate the qualified variants. Finally, the variants were obtained and the deleteriousness of variants was subsequently predicted by various bioinformatics programs (e.g. sift, polyphen2, lrt, mutationtaster, mutationassessor, fathmm, radialsvm, lr).

## Results

### General characteristics of the patients

We studied three patients with insulinoma, two female and one male. None had a family history of insulinoma. Moreover, we also excluded multiple endocrine neoplasia type 1 (MEN‐1 syndrome) from non‐tumor tissue. The WHO grading classification of pancreatic neuroendocrine tumors updated in 2010 includes neuroendocrine tumor G1, neuroendocrine tumor G2, neuroendocrine carcinoma G3 and mixed adenoneuroendocrine carcinoma 11. Depending on the classification system 12, two patients (INS1 and INS3) were classified as Grade II and one was classified as Grade I in this study. All presented with signs and symptoms of hypoglycemia. The hypoglycemia was corrected in all cases after surgical removal. General pathological and demographic characteristics for the three patients are shown in Table 1.

### Genetic analysis

Genomic DNA from insulinomas and matched blood samples was subjected to whole exome sequencing. After mapping of the human genome sequence (hg19), a total of > 86% of the exome region was covered (Table 2). Exome sequencing analysis overall identified 40 210, 40 272 and 41 910 SNVs for tumor tissue, and 41 106, 40 050 and 41 451 SNVs for the matched blood samples. We identified 55 rare somatic mutations among the three patients, of which 39 were non‐synonymous, four were nonsense and 12 were synonymous. An overview of detected somatic mutations after exome sequencing is provided in Table 3. *MEN1* gene nonsense mutations occurred in two different tumor samples. A c. 681C>G; p.Tyr227* mutation was found in exon 4 of the *MEN1* gene in INS1, and c. 346G>T; p.Glu116* mutation was found in exon 2 of the *MEN1* gene in INS2. The mutations were not present in the corresponding leukocyte DNA. Additionally, these mutations were predicted to be damaging by sift or lrt.

**Table 2:** Summary of sequencing data. C1, C2 and C3: tissues of three patients with insulinoma; N1, N2 and N3: blood of three patients with insulinoma

Sample	Reads	Read length (bp)	> *Q*_30_ (%)	Exome size (bp)	Exome coverage
C1	104 472 186	125	94.82	74 856 280	87.36%
C2	107 751 402	125	92.34	74 856 280	87.79%
C3	112 709 600	125	94.69	74 856 280	88.19%
N1	110 592 066	125	94.5	74 856 280	87.90%
N2	98 905 302	125	94.49	74 856 280	86.08%
N3	109 660 740	125	94.58	74 856 280	88.44%

**Table 3:** Overview of the gene mutations found by exome sequencing in the three patients with insulinoma

Sample	Chr	Start	Ref	Alt	Gene	Mutation type	AA change
INS1	1	1.1 × 10^8^	G	A	*PSRC1*	Nonsynonymous	p.Ala227Val
INS1	2	1 926 848	A	G	*MYT1L*	Synonymous	p.Asn231Asn
INS1	2	2.38 × 10^8^	T	C	*COL6A3*	Nonsynonymous	p.Glu1565Gly
INS1	3	1.47 × 10^8^	C	T	*ZIC4*	Nonsynonymous	p.Arg246His
INS1	3	1.89 × 10^8^	C	T	*TPRG1*	Synonymous	p.Leu120Leu
INS1	5	1.46 × 10^8^	G	T	*PPP2R2B*	Nonsynonymous	p.Pro235Thr
INS1	6	32 548 632	T	A	*HLA‐DRB1*	Nonsynonymous	p.Arg218Ser
INS1	7	1 542 657	G	A	*INTS1*	Nonsynonymous	p.Arg77Cys
INS1	7	4 249 780	T	A	*SDK1*	Nonsense	p.Leu329*
INS1	7	1.48 × 10^8^	A	G	*CNTNAP2*	Nonsynonymous	p.Lys1156Glu
INS1	7	1.57 × 10^8^	G	T	*PTPRN2*	Nonsynonymous	p.Ser827Tyr
INS1	9	99 157 190	A	G	*ZNF367*	Synonymous	p.Cys202Cys
INS1	10	99 153 502	C	A	*RRP12*	Nonsynonymous	p.Ala157 Ser
INS1	10	1.3 × 10^8^	C	G	*MKI67*	Nonsynonymous	p.Gly477Ala
INS1	11	64 575 141	G	C	*MEN1*	Nonsense	p.Tyr227*
INS1	11	1.02 × 10^8^	A	G	*CEP126*	Nonsynonymous	p.Ile674Val
INS1	13	95 887 080	G	A	*ABCC4*	Synonymous	p.Ala105Ala
INS1	14	74 968 287	G	T	*LTBP2*	Nonsynonymous	p.Pro1726Gln
INS1	16	28 943 787	G	C	*CD19*	Nonsynonymous	p.Gly70Ala
INS1	16	31 405 651	C	A	*ITGAD*	Nonsynonymous	p.Phe42Leu
INS1	17	21 318 727	A	T	*KCNJ12*	Nonsynonymous	p.Met25Leu
INS1	17	57 761 285	A	G	*CLTC*	Nonsynonymous	p.His1462Arg
INS1	19	10 799 330	G	A	*ILF3*	Nonsynonymous	p.Gly843Ser
INS2	1	22 332 008	T	C	*CELA3A*	Synonymous	p.Asp66Asp
INS2	1	40 229 393	G	A	*BMP8B*	Synonymous	p.Leu313Leu
INS2	6	32 007 839	G	T	*CYP21A2*	Nonsynonymous	p.Ala236Ser
INS2	8	52 733 231	G	A	*PCMTD1*	Nonsense	p.Arg176*
INS2	8	88 298 821	T	A	*CNBD1*	Nonsynonymous	p.Tyr322Asn
INS2	8	1.25 × 10^8^	G	A	*TMEM65*	Nonsynonymous	p.Ala179Val
INS2	8	1.25 × 10^8^	T	C	*TMEM65*	Nonsynonymous	p.Tyr176Cys
INS2	9	1.13 × 10^8^	C	A	*SVEP1*	Nonsynonymous	p.Arg902Leu
INS2	11	64 577 236	C	A	*MEN1*	Nonsense	p.Glu116*
INS2	11	89 018 006	C	A	*TYR*	Nonsynonymous	p. Pro417His
INS2	12	6 787 522	G	A	*ZNF384*	Nonsynonymous	p.Pro153Ser
INS2	12	9 243 947	A	G	*A2M*	Synonymous	p.Ser773Ser
INS2	12	1.25 × 10^8^	A	G	*UBC*	Synonymous	p.Asp647Asp
INS2	17	7 671 513	G	A	*DNAH2*	Nonsynonymous	p.Arg1290His
INS2	17	7 834 438	C	G	*TRAPPC1*	Nonsynonymous	p. Ser67Thr
INS2	17	34 797 666	G	A	*TBC1D3B*	Synonymous	p.Ala490Ala
INS2	19	41 355 849	A	G	*CYP2A6*	Synonymous	p. Leu73Leu
INS2	X	37 027 691	T	C	*FAM47C*	Nonsynonymous	p.Phe403Ser
INS3	1	12 854 188	T	C	*PRAMEF1*	Nonsynonymous	p.Cys138Arg
INS3	2	1.28 × 10^8^	G	A	*MYO7B*	Nonsynonymous	p.Gly163Asp
INS3	4	1.52 × 10^8^	C	A	*RPS3A*	Nonsynonymous	p.Asn147Lys
INS3	4	1.52 × 10^8^	A	C	*RPS3A*	Nonsynonymous	p.Gln157Pro
INS3	5	1.4 × 10^8^	T	C	*PCDHA5*	Nonsynonymous	p.Val596Ala
INS3	6	99 850 428	T	C	*PNISR*	Nonsynonymous	p.Lys439Glu
INS3	6	1.38 × 10^8^	C	T	*OLIG3*	Nonsynonymous	p.Ala175Thr
INS3	8	33 449 689	C	A	*DUSP26*	Nonsynonymous	p.Ala160Ser
INS3	10	21 903 830	T	G	*MLLT10*	Nonsynonymous	p.Cys194Gly
INS3	11	1 093 437	G	C	*MUC2*	Synonymous	p.Thr1752Thr
INS3	11	1.26 × 10^8^	C	T	*PUS3*	Nonsynonymous	p.Glu143Lys
INS3	16	7 629 904	C	T	*RBFOX1*	Synonymous	p.Asp152Asp
INS3	17	79 667 512	G	A	*HGS*	Nonsynonymous	p.Ser633Asn
INS3	19	19 030 142	G	A	*COPE*	Nonsynonymous	p.Pro6Ser

### Mutation of *MEN1* gene

**Figure 1:**
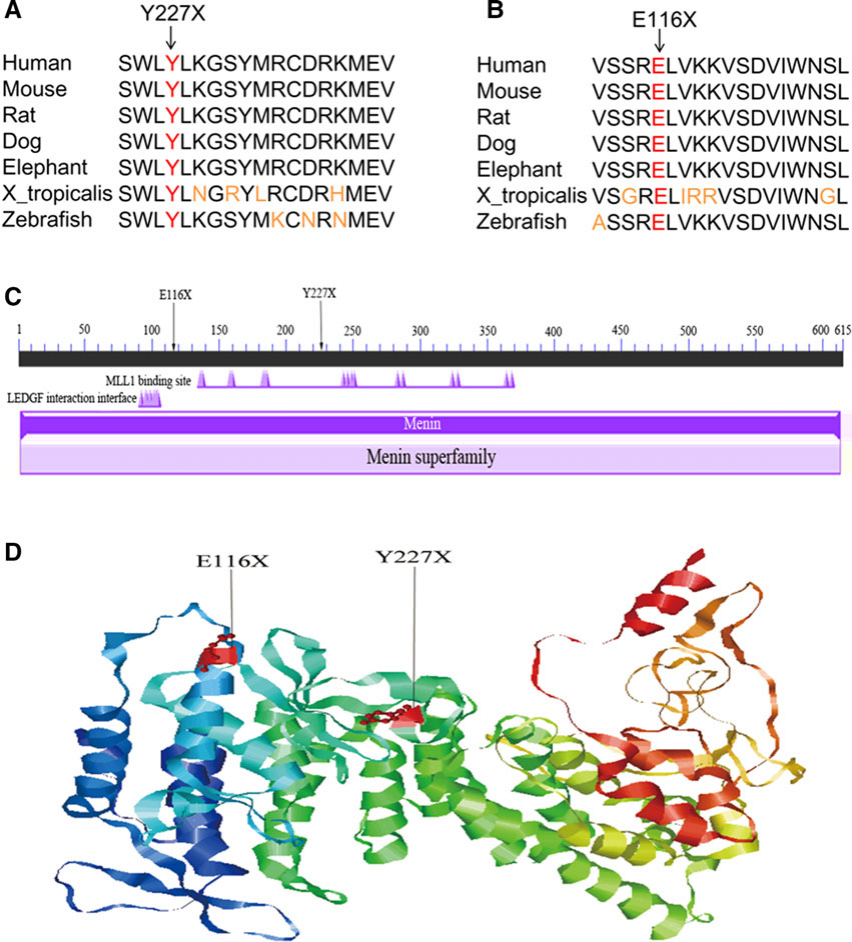
Nonsense mutation in *MEN1*. (A) Conservation of p.Tyr227* in the MEN1 protein. (B) Conservation of p.Glu116* in the MEN1 protein. (C) Locations of p.Tyr227* and p.Glu116* mutations in the conserved domain in the MEN1 protein. (D) Location of p.Tyr227* and p.Glu116* mutations on the crystal structure of the MEN1 protein.

The p.Tyr227* mutation (SWLYLKGSYMRCDRKMEV) and p.Glu116* mutation (VSSRELVKKVSDVIWNSL) both resulted in a significant truncation of menin, which may destroy the functional domain and affect its function. Amino acids of p.Tyr227* and p.Glu116* mutations are highly conserved across multiple species (Fig. 1A,B). Moreover, two nonsense mutations locate in the functional domain of *MEN1*, indicating that it may affect the binding of lysine methyltransferase 2A (Fig. 1C). In the current study, the nonsense mutations happened early in the sequence of *MEN1*, which may obviously result in a non‐functional gene product (Fig. 1D).

## Discussion

Insulinoma is a common neuroendocrine tumor with an incidence of four in every 1 million persons annually 13. Moreover, most of the insulinomas arise sporadically. Although rare, it has the potential to produce profound metabolic derangements that require early recognition and treatment. In this study, exome sequencing was performed on three sporadic insulinoma cases to delineate genetic contributors to this rare endocrine tumor. Although the overall frequency of somatic mutations was low and predicted to be damaging, there were two nonsense variants that occurred in *MEN1* in two of the three patients, namely a c. 681C>G; p.Tyr227* mutation in exon 4 of *MEN1* and a c. 346G>T; p.Glu116* mutation in exon 2 of *MEN1*. Our result showed that the mutations in *MEN1* may play an important role in the development of insulinoma. However, further functional research is needed to validate their roles.

*MEN1* consists of 10 exons 9 and encodes a protein with 615 amino acids 14; it is considered a putative tumor suppressor gene associated with neuroendocrine tumors 15. Menin, the encoded protein of *MEN1*, is a typical GTPase stimulated by nm23. It is mostly found in the nucleus and can regulate gene expression in a positive or negative way, and it has been demonstrated to interact with transcription activators, transcription repressors, cell signaling proteins and various other proteins. In addition, it plays major roles in DNA repair, cell cycle regulation and chromatin remodeling. Generally, menin is considered as a transcriptional regulator and interacts with a number of nuclear and cytosolic proteins, which indicates that it may participate in various biological pathways of tumor formation 16, 17, 18. Additionally, a number of sporadic endocrine tumors, including parathyroid adenomas, pancreatic insulinomas and pituitary prolactinomas, have somatic mutations of *MEN1* alleles, suggesting that *MEN1* may play a role in non‐hereditary endocrine tumors 15, 19, 20. Our results showed that *MEN1* mutations were found in two of the three insulinoma patients, which provided further evidence that *MEN1* might be an important factor in the pathological process of insulinoma.

It is noted that insulinomas can occur sporadically or in combination with MEN‐1 syndrome. Moreover, the *MEN1* gene is the first gene that has been identified as a candidate gene in the tumorigenesis of insulinoma. MEN‐1 syndrome represents an autosomal dominant disorder related to mutations in the *MEN1* gene mapped to chromosome 11q13 21, 22. Generally, simple and local tumor enucleation of MEN‐1 syndrome‐associated insulinomas is not likely to be curative. Although genetic testing for *MEN1* fails to detect mutation rate of 10–25%, it plays a vital role in identifying patients with hereditary insulinomas 23, 24. Therefore, genetic testing for MEN‐1 syndrome is beneficial to clinical diagnosis. Okamoto *et al*. 25 suggested that a novel six‐nucleotide insertion in exon 4 of the *MEN1* gene might contribute to the familial insulinoma. A prior study indicated that *MEN1* gene mutations were lacking in 27 sporadic insulinomas 26. By evaluating a large family with malignant insulinoma and hyperparathyroidism, Hasani‐Ranjbar *et al*. 27 found a novel *MEN1* gene frameshift germline mutation, which was associated with malignant insulinoma. Moreover, a recent study found several novel pathogenic *MEN1* mutations in sporadic cases of insulinoma 28. Herein, we found mutations in *MEN1* on chromosome 11 in patients with insulinoma, which had a damaging role in the function of the encoded protein. Our finding was consistent with other studies indicating the role of *MEN1* mutation in human sporadic insulinomas 26, 29, 30, 31, 32, which provided a crucial clue in the treatment of insulinoma. Interestingly, Waldmann *et al*. 33 found the p.E116X mutation in exon 2 of the *MEN1* gene in 21 patients with MEN‐1 syndrome and adrenal lesions. In addition, Turner *et al*. 34 identified the p.Y227X mutation in exon 4 of the *MEN1* gene in multiple endocrine neoplasia type 1. This further suggested that *MEN1* was significantly associated with insulinoma.

## Conclusions

In the current study, exome sequencing for three sporadic insulinomas identified two somatic nonsense mutations in *MEN1* (c. 681C>G; p.Tyr227* and c. 346G>T; p.Glu116*), which might induce a non‐functional gene product and contribute to the oncogenesis of sporadic insulinoma. However, there were some limitations in this study. The samples selected were small and larger samples are further needed to validate their roles in insulinoma. Further studies are needed to precisely explore the role of genetic mutations of *MEN1* in clinical manifestations of these patients. In addition, validation of *MEN1* mutations (such as by Sanger sequencing) and study of the underlying biological function of the *MEN1* mutations is needed in a further study.

## Author contributions

JD, QS and MW analyzed and interpreted the data. CQ was the major contributor in writing the manuscript. CY designed the project. All authors read and approved the final manuscript.
